# Case report: A finding of PVOD and PAH in first degree relatives suggests shared heritable risk and overlapping features of both pulmonary vascular diseases

**DOI:** 10.1002/rcr2.70064

**Published:** 2024-11-25

**Authors:** Roger Winters, Lindsay M. Forbes, Dunbar Ivy, Carlyne Cool, Bryan D. Park, Peter Hountras, David Badesch, Sue Gu, Edda Spiekerkoetter, Roham Zamanian, Stacey LeierGluck, Todd M. Bull

**Affiliations:** ^1^ Internal Medicine Residency Program, Department of Medicine University of Colorado Aurora Colorado USA; ^2^ Division of Pulmonary Sciences & Critical Care Medicine, Pulmonary Vascular Disease Center University of Colorado Aurora Colorado USA; ^3^ Division of Pediatric Cardiology University of Colorado Children's Hospital Aurora Colorado USA; ^4^ Division of Pathology University of Colorado Colorado USA; ^5^ Division of Pulmonary, Allergy & Critical Care Medicine Stanford University Stanford California USA

**Keywords:** genetics, pulmonary circulation and pulmonary hypertension, PVOD or pulmonary veno‐occlusive disease, rare lung diseases

## Abstract

Pulmonary veno‐occlusive disease (PVOD) is a rare form of pulmonary vascular disease that is difficult to distinguish clinically from pulmonary arterial hypertension (PAH). Multiple genes have been implicated in disease pathogenesis in PAH and PVOD and the diseases are thought to be genetically distinct. In this report we present a case of first‐degree relatives with pathological evidence of PVOD and PAH. The index patient was diagnosed with PAH at age 42, was treated with escalating pulmonary vasodilator therapy, but eventually succumbed to her disease. On autopsy, her pathology was consistent with PAH. Her son was diagnosed with PAH at age 16, did well on pulmonary vasodilator therapy for over 10 years, but ultimately developed refractory right ventricular failure and received a heart and lung transplantation. Pathology of his explanted lung was consistent with PVOD, and genetic testing was negative for recognized variants that cause PAH or PVOD.

## Graphical abstract

We present a rare case of first‐degree relatives with pathologically proven PVOD and PAH. Genetic testing was negative for recognized variants that cause PAH or PVOD.

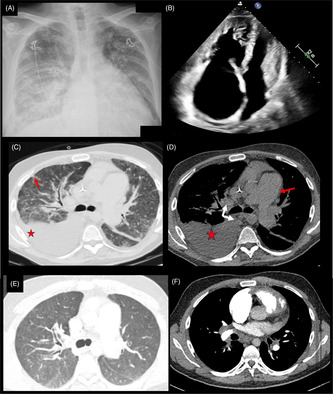

## INTRODUCTION

Pulmonary veno‐occlusive disease (PVOD) is a rare form of pulmonary vascular disease that is difficult to distinguish clinically from pulmonary arterial hypertension (PAH).^1^ However, the response to pulmonary vasodilators is more variable and survival is significantly lower in PVOD.^2^, ^3^, ^4^ The differential response to therapy arises from PVOD's unique biological features, including preferential involvement of the pulmonary venous system.^5^ This venous involvement is associated with capillary damage and post capillary remodelling.^6^ While lesions predominate at the post‐capillary level, PVOD frequently involves lesions of the capillaries, arteries and veins.^7^

While the histological features of PVOD are distinct, lung biopsy is often prohibitively high‐risk and the diagnosis must be suspected based upon clinical clues including significant reduction in the diffusing capacity for carbon monoxide (DLCO), mediastinal lymphadenopathy, ground‐glass opacities, pleural effusions, and interlobular septal thickening.^1^, ^2^, ^8^

Multiple genes have been implicated in disease pathogenesis in PAH and PVOD and the diseases are thought to be genetically distinct.^1^ In this report we present the a case of first‐degree relatives with pathological evidence of PVOD and PAH.

## CASE REPORT

**FIGURE 1:**
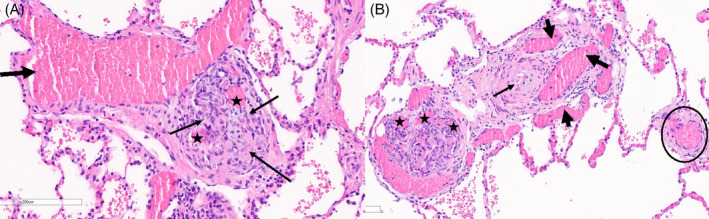
Lung tissue pathology from index patient. A/B: Pathology from index patient's lung tissue showing: (A) complex intraluminal proliferation of vascular cells (thin arrows) within the branch point of a small pulmonary artery. These precapillary plexiform lesions are histologic hallmarks of severe pulmonary arterial hypertension. The residual irregular lumina are highlighted (stars). The lumen of the “parent” artery is indicated by the thick arrow. (B) Small pulmonary artery with a glomeruloid proliferation of intraluminal vascular cells intermixed and surrounded by the slit‐like vascular spaces (stars). The upper right portion of the pulmonary artery shows concentric intimal proliferation (thin arrow) as well as aneurysmal dilatation (thick arrows). Note the normal small pulmonary artery at the right (circle).

The index patient was diagnosed with PAH at age 42. She was treated with bosentan with clinical improvement. Unfortunately, several years later she had significant clinical worsening and despite escalation to epoprostenol therapy, she expired from right ventricular (RV) failure. Her autopsy revealed severe plexiform pulmonary arteriopathy consistent with PAH without pulmonary venous involvement (Figure 1A,B).

**FIGURE 2:**
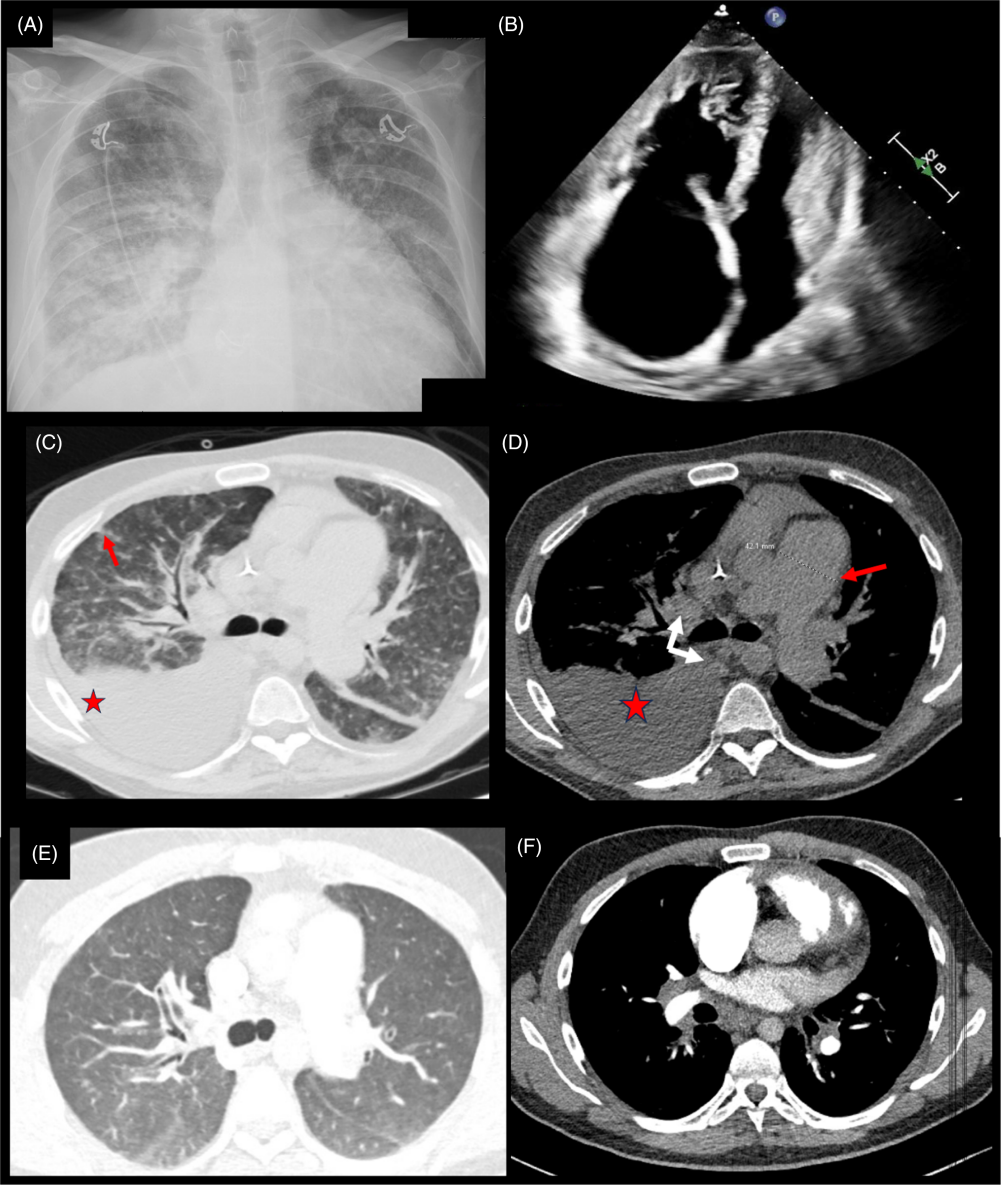
Chest x‐ray, transthoracic echocardiogram, and CT imaging from patient on admission for refractory right ventricular failure and during period of clinical stability. (A) Chest x‐ray on admission for volume overload and RV failure showing diffuse interstitial and alveolar opacities and cardiomegaly. (B) Apical 4‐chamber view on echocardiography showing flattened septum consistent with RV volume and pressure overload, and severe right atrial dilation. (C, D) High‐resolution CT Chest prior to lung transplantation, with (C) lung windows showing ground‐glass opacities, presence of septal thickening (red arrow), and (D) mediastinal windows showing severely enlarged pulmonary artery (red arrow), mediastinal lymphadenopathy (white arrows), and large right‐sided pleural effusion (red star). E/F: On CT Chest from six years prior to admission, patient had revealed (E) diffuse heterogeneous ground glass opacities, centrilobular nodularity with evidence of septal thickening, evidence of pulmonary venous extension to the periphery of the lung fields with tortuosity, and (F) substantial hilar lymphadenopathy.

**FIGURE 3:**
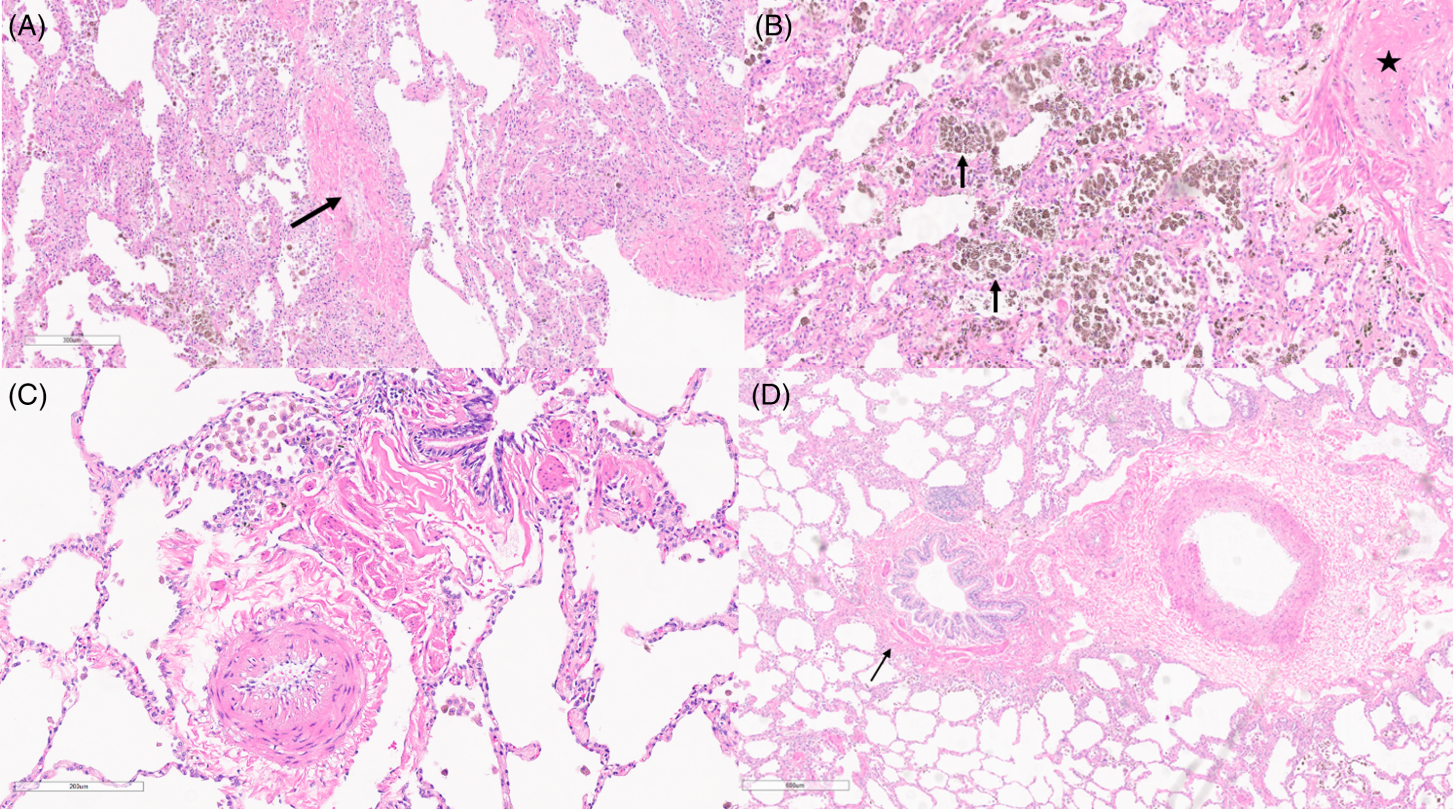
Lung tissue pathology from index patient's son: A/B: Pathology from patient's son's explanted lung tissue showing (A) fibrous occlusion of a septal vein (arrow) consistent with PVOD and (B) hemosiderin laden macrophages which are another common finding in PVOD. The vessel at the upper right shows luminal occlusion (star). C/D: Additional pathological images are the additional pictures at (C) 200 um and (D) 600 um showing minimal arterial involvement aside from thickened pulmonary artery (arrow) and a notable lack of plexiform lesions.

Her son was initially diagnosed with PAH at age 16 (Table 1). No other family members have been affected by pulmonary vascular disease. The patient's son and his mother were thoroughly worked up for other causes of PAH including environmental exposures, autoimmune diseases, and toxin exposures. Based on his mother's history of PAH, he was diagnosed with heritable PAH, but genetic testing was negative for recognized PAH and PVOD variants including *BMPR2* and *EIF2AK4* (Table 2). He was treated with oral pulmonary vasodilators and was stable on therapy for 16 years (Table 1). He then developed RV failure and was initiated on intravenous treprostinil (Table 1). His clinical status further declined; he was re‐admitted for refractory RV failure and volume overload (Figure 2A). Trans thoracic echocardiography (TTE) showed a flattened septum consistent with RV overload, severely decreased RV function, a moderate pericardial effusion, normal left ventricular ejection fraction (LVEF), and no evidence of diastolic function (Figure 2C). Notably, the patient had a normal LVEF and lack of diastolic dysfunction on TTE throughout his clinical course. CT revealed ground‐glass opacities, interlobular septal thickening, plural effusions, pulmonary edema, and mediastinal lymphadenopathy (Figure 2C,D). Prior CT from 6 years prior to the admission also showed ground glass opacities, centrilobular nodularity with evidence of septal thickening, evidence of pulmonary venous extension to the periphery of the lung fields with tortuosity, and substantial hilar lymphadenopathy (Figure 2E,F). Ultimately, the patient underwent heart and lung transplant and has done well since transplantation. Pathology from the patient's explanted lung revealed fibrinous occlusion of septal veins and hemosiderin laden macrophages, consistent with PVOD (Figure 3A,B) The explanted lung tissue lacked plexiform lesions or significant arterial involvement aside from thickening of the pulmonary arteries (Figure 3C,D).

**TABLE 1:** RHC, serological, and functional class data during course of treatment with corresponding medications.

Clinical data	Patient age: 2004/16 y/o	2005/17	2007/19	2009/21	2013/25	2017/29	2021/33
Right atrial pressure (mmHg)	6	6	7	8	8	11	17
Mean pulmonary artery pressure (mmHg)	60	64	33	40	48	54	80
Pulmonary vascular resistance (WU)	8.29	11.2	2.23	3.3	8.8	7.7	12.6
Pulmonary arterial wedge pressure (mmHg)	7	7	18	8	5	10	4
Cardiac output (L/min)^a^	6.39	5.08	6.56	9.69	6.08	3.11	3.09
Cardiac index (L/min/m^2^)^a^	3.4	2.58	3.35	4.82	3.2	1.54	1.6
B‐type natriuretic peptide (pg/mL)		22	46		142	516	553
6 Minute Walk Distance (m)	488	537	618	585	624	597	174
New York Heart Association Functional Class	II	II	I	II	II	I	III
Medications	None	Sitaxsentan Warfarin	Sitaxsentan Iloprost Sildenafil Warfarin	Sildenafil Ambrisentan Warfarin	Tadalafil Macitentan Warfarin	Tadalafil Ambrisentan Selexipag Warfarin	Tadalafil Ambrisentan IV Treprostinil Warfarin

^a^ By thermodilution.

**TABLE 2:** Genetic testing of the patient's son was negative for recognized PAH/PVOD variants.

Gene	Pathological variant identified
*ACVRL1*	No
*AQP1*	No
*ATP13A3*	No
*BMPR1B*	No
*BMPR2*	No
*CAV1*	No
*EIF2AK4*	No
*ENG*	No
*GDF2*	No
*KCNA5*	No
*KCNK3*	No
*SMAD9*	No
*SOX17*	No
*TBX4*	No

## DISCUSSION

We believe this is a novel report of pathological evidence of PVOD and PAH in first‐degree relatives. The mother's pathology showed classic pre‐capillary plexiform lesions without evidence of pulmonary vein involvement (Figure 2A,B). However, the son's explanted lung tissue revealed PVOD with fibrinous occlusion of septal veins (Figure 2C,D). Interestingly, he behaved clinically like PAH for 16 years with an excellent clinical response to pulmonary vasodilators until the year prior to transplant. The clinical time course is atypical of PVOD, but serial echocardiograms lacked evidence of left ventricular systolic or diastolic dysfunction, the patients PCWP pre‐transplant was indicative of normal left sided filling pressures, and his pathology lacked significant pulmonary arterial involvement.

The presence of PAH and PVOD within the same pedigree introduces the consideration of a “crossover” genetic variant which predisposes carriers to two unique phenotypes of pulmonary vascular disease. These diseases have genetic underpinnings which are typically thought of as unique. Most identified genetic variants in PAH cause disease through autosomal dominant inheritance with variable penetrance, whereas heritable PVOD arises from autosomal recessive, biallelic mutations in *EIF2AK4*.^9^ In PVOD, the prevalence of biallelic *EIF2AK4* mutations was estimated at 29%.^2^ Meanwhile, the prevalence of disease‐associated variants in patients with heritable PAH is 70%–87%, with mutations of genes in the bone morphogenetic protein receptor type 2 (*BMPR2*)/transforming growth factor‐β pathway predominating.^9^ *EIF2AK4* loss‐of‐function mutations may also affect the *BMPR2* cellular cascade through modulation of Tribbles Homologue 3.^10^ In PAH, GCN2, the protein coded by the *EIF2AK4* gene is also decreased.^11^ Thus, heritable PVOD and PAH may have overlapping pathogenic mechanisms.^12^ Additionally, homozygous *EIF2AK4* variants have been identified with increased prevalence in patients with PAH, and *BMPR2* variants have been identified in patients with PVOD.^9^, ^13^ However, our patient demonstrated no variants in genetic testing for known PAH/PVOD associated genes and thus, this case raises the possibility of a novel genetic variant associated with both PAH and PVOD phenotypes.

Some patients initially receive a diagnosis of PAH which is later revised to PVOD based upon imaging or response to therapy.^1^, ^2^ It is possible that this reflects evolution of vascular remodelling and disease characteristics from a state that is PAH‐like to one that is recognized as PVOD. Severe forms of PAH, including those associated with *BMPR2* mutations can have venous involvement and PVOD is known to have a component of arterial involvement.^6^, ^11^ Muscular remodelling of pulmonary veins is more extensive in PAH patients carrying a *BMPR2* mutation than other forms of PAH.^14^

A prior report presented a case of hereditary PVOD who had pulmonary arterial remodelling on lung biopsy, and years later after developing clinical pulmonary hypertension, had pathology consistent with PVOD.^15^ Our patient's years'‐long history of PAH with a stable clinical course followed by acute progression of disease supports the possibility of a similar underlying shift in the disease physiology from one that was PAH‐like to PVOD‐like.

In summary, this case report presents first‐degree relatives with pathological evidence of PVOD and PAH. This case raises the question of whether a novel mechanism underlies shared pathogenesis of PVOD and PAH.

## AUTHOR CONTRIBUTIONS

Drs. Winters, Bull, and Forbes conducted background research and analysis, and drafted and revised this manuscript. Dr. Ivy provided data from the patient's paediatric visits and revised the manuscript. Dr. Cool analysed the pathological specimens and provided revisions to the manuscript. Stacey LeierGluck helped coordinate with the patient and pathology departments. Drs Park, Hountras, Gu, Badesch, Spiekerkoetter, and Zamananian were involved with the care of the patient and provided revisions to the manuscript.

## FUNDING INFORMATION

This research received no specific grant from any funding agency in the public, commercial, or not‐for‐profit sectors.

## CONFLICT OF INTEREST STATEMENT

None declared.

## ETHICS STATEMENT

The authors declare that appropriate written informed consent was obtained for the publication of this manuscript and accompanying images.

## Data Availability

The data that support the findings of this study are available from the corresponding author upon reasonable request.
